# Clinical predictors of pathological good response in locally advanced rectal cancer

**DOI:** 10.1186/s13014-020-01741-x

**Published:** 2021-01-13

**Authors:** Kongfeng Shao, Rong Zheng, Anchuan Li, Xiaobo Li, Benhua Xu

**Affiliations:** 1 Department of Radiation Oncology, Fujian Medical University Union Hospital, No.29 Xinquan Road, Gulou District, Fuzhou, 350001 People’s Republic of China; 2 College of Medical Technology and Engineering, Fujian Medical University, Fuzhou, People’s Republic of China; 3 School of Clinical Medicine, Fujian Medical University, Fuzhou, People’s Republic of China; 4 College of Union Clinical Medicine, Fujian Medical University, Fuzhou, People’s Republic of China

**Keywords:** Locally advanced rectal cancer, ypT0–1N0, Pathological good response, Predictors

## Abstract

**Purpose:**

The aim of this study was to identify the clinical predictors of pathological good response (PGR) after neoadjuvant chemoradiotherapy (nCRT) in locally advanced rectal cancer (LARC) to clarify the indications for local excision.

**Methods and materials:**

A total of 173 patients with LARC (cT3–4/N +) who were treated with nCRT followed by surgery were enrolled in our retrospective study. Patients were categorized into two groups according to the different tumor responses of surgical pathology. Stage ypT0–1N0 was defined as the group with PGR, and stage ypT2–4N0/ypTanyN + was the defined as the pathological poor response (PPR) group, and the potential predictors were compared.

**Results:**

Of 173 patients, PGR was achieved in 57 patients (32.95%). The distance from the inferior margin of the tumor to the anal verge, cT classification, pretreatment carcinoembryonic antigen (CEA) and the interval from the end of radiation to surgery were correlated with pathological response. In the multivariate analysis, the distance from anal verge < 5 cm (OR = 0.443, p = 0.019), pretreatment CEA < 5 ng/mL (OR = 0.412, p = 0.015) and the interval from the end of radiation to surgery ≥ 84 days (OR = 2.652, p = 0.005) were independent predictors of PGR.

**Conclusions:**

The distance from the inferior margin of the tumor to the anal verge, pretreatment CEA and the interval from the end of radiation to surgery were significant predictors of PGR in LARC. A prospective study is needed to further validate these results in the future.

## Introduction

Since the results of the phase III clinical trial (CAO/ARO/AIO-94) comparing the timing of concurrent chemoradiotherapy were published [1], preoperative fluorouracil-based neoadjuvant chemoradiotherapy (nCRT) followed by total mesorectal excision (TME) combined with postoperative adjuvant chemotherapy has become the standard treatment for locally advanced rectal cancer (LARC).

Radical surgery may cause morbidity and various forms of functional impairment, such as defecation [2, 3], urinary [4] and sexual dysfunction [5]. At the same time, some surgeons selected local excision (LE) rather than TME for patients who responded well to nCRT to preserve organs and improve the quality of life after operation. A retrospective multicenter study reported that patients with LE alone had a better quality of life and bowel function than those who underwent TME or LE followed by TME [6]. The CARTS study also found an improved emotional functioning score for patients undergoing transanal endoscopic microsurgery according to the QLQ-C30 questionnaire [7].

Additionally, it has been reported that LE could provide acceptable oncological outcomes among individuals who responded well to nCRT. A phase II multicenter trial in Italy showed that LE is a good option for patients with a major clinical response after nCRT, and the 3-year overall survival (OS), disease-free survival (DFS), and local disease-free survival were 91.5%, 91.0% and 96.9%, respectively [8]. A propensity score analysis used ypT0–1 rectal cancer to match groups (LE:TME = 1:1) and found that LE did not increase the tumor recurrence rate compared with TME (4.8% vs 7.14%, p = 0.646), and the two groups had similar 5-year OS (96.6% vs 88.0%, p = 0.238) and DFS (95.2% vs 91.6%, p = 0.33) [9]. However, patients with stage ypT2 turned out to have high risk of local failure and poor survival when treated with LE; therefore, it cannot be justified as an indication for LE [10–12].

Consequently, identifying LARC patients with stage ypT0–1N0 disease before surgery is of great clinical significance. However, there are still limitations in terms of ways to evaluate the extent of primary tumor regression after nCRT, as both magnetic resonance imaging (MRI) and endoscopy show high specificity but poor sensitivity [13, 14]. The GRECCAR 2 trial assessed tumor regression by digital rectal examination, enteroscopy and MRI, and with thorough preoperative examination, one-third of patients still had ypT2–3/R1 tumors and underwent salvage TME surgery, which increased morbidity and side effects more than those who had LE alone (p = 0.0001) [15]. Moreover, stage ypN0 affects the clinical decision-making of LE, and existing assessment methods have difficulty providing accurate regional lymph node involvement [16, 17].

Therefore, it is expected to identify the clinical predictors of stage ypT0–1N0 in LARC in addition to routine examinations to clarify the indications for LE, thus preserving rectal and adjacent organ function.

## Methods and materials

### Patients

This study retrospectively analyzed a total of 173 patients with LARC who were treated with nCRT followed by surgery at our institution between August 2018 and October 2019. The inclusion criteria were as follows: (1) pathologically confirmed rectal adenocarcinoma; (2) stage II-III (cT3–4/N +) by MRI or computed tomography (CT) combined with endorectal ultrasound according to the eighth edition of the American Joint Committee on Cancer (AJCC) Staging Manual; (3) no history of either prior surgery, pelvic radiotherapy or systematic chemotherapy; and (4) Eastern Collaborative Oncology Group (ECOG) performance status score of 0–1 and no other serious complications. Patients with other malignant tumors were excluded.

### Neoadjuvant chemoradiotherapy

Two chemotherapeutic regimens with dosages were given as follows: (1) Capox: oxaliplatin 130 mg/m^2^ intravenously guttae day 1, capecitabine 825 mg/m^2^ twice daily oral days 1–14, every 3 weeks, for 2 cycles during concurrent radiotherapy; another 2 cycles were performed during the interval from the end of radiation to surgery; (2) capecitabine alone: capecitabine 825 mg/m^2^ twice daily oral, during the whole period of radiotherapy; another 1 cycle increased dosages to 1250 mg/m^2^ was performed in 2 weeks during the waiting period.

The gross tumor volume (GTV) was calculated based on clinical information, including digital rectal examination, endoscopy ultrasound, and abdominopelvic MRI. The clinical target volume (CTV) included all mesorectum, presacral soft tissue, obturator and internal iliac lymphatic drainage regions. The planning target volume (PTV) was defined as the GTV or CTV with uniform margins of 10 mm. The neoadjuvant radiotherapy regimens consisted of 3-dimensional conformal radiotherapy (3D-CRT) and intensity-modulated radiation therapy (IMRT). A dose of 50.4 Gy was delivered to PTV-GTV with 3D-CRT in 28 fractions, while 50 Gy was delivered with IMRT in 25 fractions. 45 Gy was delivered to PTV-CTV in 25 fractions for both types of regimens.

### Surgery and pathology

Surgery was performed strictly according to the principle of TME by experienced surgeons. Patients with a good tumor response after nCRT underwent LE if they so desired. The types of surgical procedures included Miles, Dixon, Hartmann and LE. Pathological complete response (pCR) was defined as no residual tumor cells in the resected specimens, including lymph nodes, under a microscope (ypT0N0).

### Efficacy evaluation

In this study, downstaging was defined as a pathological stage lower than the clinical stage by pretreatment imaging evaluation. Patients were categorized into two groups according to the different tumor responses of surgical pathology: stage ypT0–1N0 was defined as the group with pathological good response (PGR), and stage ypT2–4N0/ypTanyN + was the pathological poor response (PPR) group. The following potential predictors were evaluated: gender, age, distance from the inferior margin of the tumor to the anal verge, clinical and pathologic TNM stage, levels of carcinoembryonic antigen (CEA) and carbohydrate antigen 199 (CA199), circumferential resection margin (CRM), extramural vascular invasion (EMVI), type of chemotherapy, interval from the end of radiation to surgery and surgical approach.

### Data analysis

Descriptive statistics are reported as the median and range. The association between different tumor responses and clinicopathological parameters was evaluated using the Pearson Chi-square test or Fisher exact test. Characteristic parameters with a *p *value < 0.050 were selected as potentially relevant predictor variables and were entered into a multivariable logistic regression analysis for PGR by using the backward method, and the receiver operating characteristic (ROC) curve was generated to evaluate the efficacy of the model. A *p *value < 0.050 was considered statistically significant. All the data were analyzed with SPSS 22.0 (SPSS Inc., Chicago, IL, USA).

## Results

### Patients’ characteristics

A total of 173 patients with LARC who were treated with nCRT followed by surgery were enrolled; 103 patients were male, and 70 patients were female, with a median age of 56 (range 24–79 years). The median distance from the anal verge was 5.3 cm (range 1–13.2 cm), and the median interval from the end of radiation to surgery was 78 days (range 46–227 days). Baseline characteristics are detailed in Table 1.

**Table1:** Characteristics of locally advanced rectal cancer patients

Characteristics values	Counts
Gender	
Male	103
Female	70
Age (years, median [range])	56 (24–79)
The distance from anal verge (cm, median [range])	5.3 (1–13.2)
cT classification	
2	10
3	69
4	94
cN classification	
0	7
1	44
2	122
Clinical stage	
II	7
III	166
Pretreatment CEA (ng/mL, median [range])	4 (0.6–306.6)
Pretreatment CA199 (U/mL, median [range])	13.01 (0.75–1000)
Posttreatment CEA (ng/mL, median [range])	2.5 (0.6–71.8)
Posttreatment CA199 (U/mL, median [range])	11.66 (0–107.2)
Concurrent chemotherapy	
Capecitabine	78
Capox	95
CRM	
Positive	80
Negative	93
EMVI	
Positive	86
Negative	87
The neoadjuvant–surgery interval (day, median [range])	78 (46–227)
The types of surgical procedures	
Dixon	152
Miles	10
Hartmann	2
Local excision	9

Clinical TNM stage, according to the eighth edition of the AJCC staging manual

*CEA* carcinoembryonic antigen, *CA199* carbohydrate antigen 199, *CRM* circumferential resection margin, *EMVI* extramural vascular invasion

### Postoperative pathological features

The rates of ypT downstaging, ypN downstaging and yp total stage downstaging were 80.35%, 83.82% and 73.41%, respectively. There were 47 patients (27.17%) who achieved pCR. Furthermore, 57 patients (32.95%) achieved PGR, while 116 patients (67.05%) achieved PPR. The rate of R0 resection was 99.42%, only one case showed microscopic residual tumor at the lower margin, and the rate of anal preservation was 93.06% (Table 2).

**Table 2:** Postoperative pathological features

Pathological characteristics	Counts
ypT classification	
0	48
1	11
2	39
3	71
4	4
ypN classification	
0	129
1	32
2	12
T classification downstaging (cT > ypT)	
Yes	139
No	34
N classification downstaging (cN > ypN)	
Yes	145
No	28
Total stage downstaging (cStage > ypStage)	
Yes	127
No	46
Pathological stage	
pCR (ypT0N0)	47
I	43
II	39
III	44
Tumor response	
Stage ypT0–1N0	57
Stage ypT2–4N0/ypTanyN +	116
Surgical margin	
R0	172
R1	1
Anal preservation	
Yes	161
No	12

Pathological TNM stage, according to the eighth edition of the AJCC staging manual

*pCR* pathological complete response

### Characteristic parameters with tumor response

The distance from the inferior margin of the tumor to the anal verge (p = 0.007), cT classification (p < 0.001), levels of pretreatment CEA (p = 0.006), and the interval from the end of radiation to surgery (p = 0.002) were significantly correlated with tumor response in the univariate Chi-square analysis when excluding cN classification, chemotherapeutic regimens, imaging features and the types of surgical procedures (Table 3).

**Table 3:** Characteristic parameters with tumor response

Variables	Classification	Tumor response		p
		ypT0–1N0	ypT2–4N0/ypTanyN +	
Gender				0.523
	Male	32	71	
	Female	25	45	
Age (years)				0.237
	< 60	29	70	
	≥ 60	28	46	
The distance from anal verge (cm)				0.007
	< 5	33	42	
	≥ 5	24	74	
cT classification				< 0.001
	2	9	1	
	3	16	53	
	4	32	62	
cN classification				0.513
	0	3	4	
	1	17	27	
	2	37	85	
Clinical stage				0.874
	II	3	4	
	III	54	112	
Pretreatment CEA (ng/mL)				0.006
	< 5	41	58	
	≥ 5	16	58	
Posttreatment CA199 (U/mL)				0.799
	< 37	52	106	
	≥ 37	5	10	
Pretreatment CEA (ng/mL)				0.690
	< 5	49	97	
	≥ 5	8	19	
Posttreatment CA199 (U/mL)				0.735
	< 37	54	110	
	≥ 37	3	6	
Concurrent chemotherapy				0.127
	Capecitabine	21	57	
	Capox	36	59	
CRM				0.594
	Positive	28	52	
	Negative	29	64	
EMVI				0.450
	Positive	26	60	
	Negative	31	56	
The neoadjuvant–surgery interval (day)				0.002
	< 84	25	79	
	≥ 84	32	37	
The types of surgical procedures				0.303
	Dixon	48	104	
	Others	9	12	

Clinical TNM stage, according to the eighth edition of the AJCC staging manual

*CEA* carcinoembryonic antigen, *CA199* carbohydrate antigen 199, *CRM* circumferential resection margin, *EMVI* extramural vascular invasion

### Clinical predictors and predictive model of PGR

Backward selection was employed to exclude the cT classification with no bearing on significance. A distance from the anal verge < 5 cm (OR = 0.443, p = 0.019), pretreatment CEA < 5 ng/mL (OR = 0.412, p = 0.015) and an interval from the end of radiation to surgery ≥ 84 days (OR = 2.652, p = 0.005) were considered as clinical predictors of PGR (Table 4).

**Table 4:** Logistic regression analysis with stage ypT0–1N0 as dependent variable

Variables	Regression coefficient	SE	*p *value	Odds ratio (95%CI)
The distance from anal verge	− 0.813	0.346	0.019	0.443 (0.225–0.873)
Pretreatment CEA	− 0.887	0.363	0.015	0.412 (0.202–0.84)
The neoadjuvant–surgery interval	0.975	0.347	0.005	2.652 (1.345–5.23)
Constant	− 0.365			

*SE* standard error, *CI* confidence interval, *CEA* carcinoembryonic antigen

**Fig. 1:**
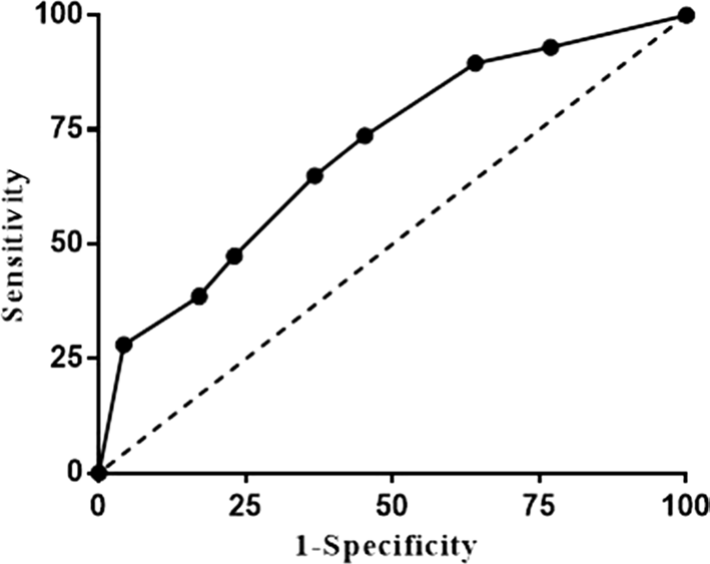
The receiver operating characteristic curve was generated to evaluate the logistic regression model, and the area under the curve was 0.702, which indicates moderate discriminative performance of this predictive model

Taking tumor response as the variable of state, the results of the logistic regression model in Table 4 were assessed. The area under the curve (AUC) was 0.702, which indicates moderate discriminative ability in this model (Fig. 1).

### Risk factors for PGR

**Fig. 2:**
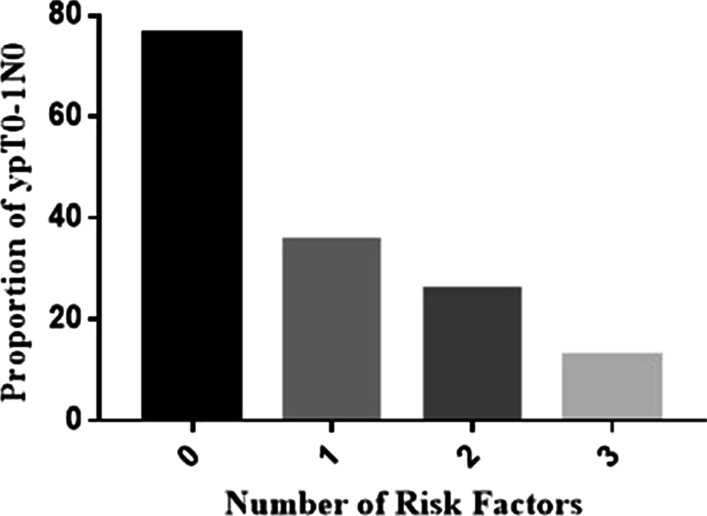
The proportions of stage ypT0–1N0 peaked at 76.19% with no risk factors, which included a distance from the anal verge ≥ 5 cm, pretreatment CEA ≥ 5 ng/mL and the neoadjuvant-surgery interval < 84 days, followed by a downward trend with increased risk factors

Based on the above results, a distance from the inferior margin of the tumor to the anal verge ≥ 5 cm, pretreatment CEA ≥ 5 ng/mL and an interval from the end of radiation to surgery < 84 days were recorded as three risk factors for poor tumor response. The proportions of PGR in the corresponding population with different risk factors were as follows: no risk factor, 76.19% (16/21); 1 factor, 35.59% (21/59); 2 factors, 25.81% (16/62); and 3 factors, 12.90% (4/31) (p < 0.001). The proportion of PGR in patients without risk factors was significantly higher than that in all others with at least one risk factor (Fig. 2).

## Discussion

With the development of surgical technology, patients with early rectal cancer who underwent LE were found to have no significant difference in survival compared with those treated with TME [18, 19]. This finding was also confirmed in patients with LARC who responded well to nCRT [8–10]. Therefore, this study used clinical data to screen out the relevant predictors of stage ypT0–1N0 after nCRT in LARC to guide individualized treatment strategies.

A retrospective study of 562 patients demonstrated that a distance from the anal verge > 5 cm was associated with a lower tumor downstaging rate [20]. Proximity to the anal verge is one of the favorable predictors for tumor response in another large retrospective cohort [21]. Moreover, our results indicated that a distance from the anal verge < 5 cm was a predictor for stage ypT0–1N0 in the multivariable analysis. Conversely, Han’s research [22] found that a moderate tumor distance (6–10 cm) was an independent predictive factor for pCR; other studies have also reported similar results [23, 24]. Different tumor locations showed divergent responses in patients with LARC who were treated with nCRT. The association of tumor location and response to chemoradiation is also unclear. The possible explanations were that lower tumors could receive a better treatment dose due to the fixed position, and concerns with small bowel toxicity in higher tumors could affect treatment planning.

CEA is one of the most widely used and readily available tumor markers for the management of colorectal cancer. Probst et al. [25] screened out 18,113 patients with LARC by selecting from the 2006–2011 National Cancer Data Base, 47% had elevated pretreatment CEA which was significantly associated with decreased pCR (OR = 0.65, p < 0.001), pathological tumor regression (OR = 0.74, p < 0.001) and downstaging (OR = 0.77, p < 0.001). A CEA level ≤ 5 ng/ml was a significant predictor of downstaging (OR = 16.0, p = 0.014) and was significantly associated with downsizing (> 60%, p = 0.012) in Yeo’s study results [26]. A case-matched control study of KROG 14–12 [27] also reported that pretreatment CEA > 5 ng/mL is a negative predictor of tumor downstaging. This is also consistent with our results, which supported that pretreatment CEA < 5 ng/mL could be a considerable clinical predictor of stage ypT0–1N0 in LARC.

Investigations regarding the best interval from the end of radiation to surgery began to appear as early as the 1990s, the most famous of which was the Lyon R90-01 randomized trial [28]. It was generally accepted that the interval should be extended to 6–8 weeks due to the long interval group that showed a better pathologic response. Another phase II clinical trial to investigate extending the interval and administering additional mFOLFOX-6 during the waiting period found that the 11-week group showed a modest increase in the pCR rate without increasing complications [29]. When the mean interval time reached 19.3 weeks, the pCR rate was as high as 38% [30]. However, it did not seem to obviously improve the tumor response as the interval time increased blindly. Rombouts et al. [31] retrieved 1073 LARC patients from the population-based Netherlands Cancer Registry between 2006 and 2011, and the highest proportion of patients with stage ypT0–1N0 was 26.6% when the treatment interval ranged from 11–12 weeks. Sloothaak et al. [32] also observed that the proportion of stage ypT0–1N0 peaked at 23.2% with 10–11 week intervals, followed by a downward trend. Interestingly, our study proved that an interval ≥ 84 days (OR = 2.652, p = 0.005) was an independent predictor of stage ypT0–1N0, and there were no significant differences in the quality of surgery or postoperative complications over time.

It is worth noting that a variety of preoperative examinations, such as digital rectal examination, endoscopy and pelvic MRI, can be used to rigorously assess the primary tumor response. In particular, the preoperative diagnosis of the status of regional lymph nodes is extremely dependent on imaging. Kim et al. [33] found that the probability of lymph node metastasis was correlated with ypT classification. Positive lymph nodes were detected in 3.4% of ypT0–1 patients, 16.9% of ypT2 patients, 49.3% of ypT3 patients and 42.9% of ypT4 patients. Our findings also supported Kim’s idea; namely, positive lymph nodes were detected in 2 of 51 (3.92%) ypT0–1 patients and in 6 of 38 (15.79%) ypT2 patients. It was considered that lymph node metastasis was rare in ypT0–1 patients. In addition, 18-FDG-positron-emission tomography integrated with computed tomography (18-FDG-PET/CT) could prompt a higher metabolic profile of disease in the worse tumor regression [34], which could be useful to guide the choice of LE in LARC.

Previously a multicentric study in Italy indicated that radiation dose intensification (range 52.5–57.5 Gy) appeared feasible, safe and effective in terms of pathological response [35]. Of which people that underwent LE, a month later, did not report any postoperative complications. More recently, a prospective observational study mentioned that radiation dose intensification, delivered 60 Gy in 30 fractions, showed a better pathologic response with acceptable toxicity related to nCRT in T3 tumors [36]. A longer follow-up period is warranted. Notably, some potential factors may provide a higher likelihood for the choice of LE in LARC and deserve further investigation.

Nevertheless, there are still some limitations in our study. First, the data were derived from a single institution, and insufficient samples may lead to the failure of some clinically related factors, such as cT classification, to show significant differences. Second, the characteristic parameters included were not comprehensive enough, which may lead to a decrease in the efficiency of the model. Finally, we used a multivariable logistic regression model but lacked an independent validation cohort to confirm the value of the above predictors. Further studies should expand the sample and introduce more variables to improve the effectiveness of the model to stratify and guide patients for individualized treatment strategies, especially for LE management after nCRT in LARC.

## Conclusion

In our retrospective study, a distance from the inferior margin of the tumor to the anal verge < 5 cm, pretreatment CEA < 5 ng/mL and the interval from the end of radiation to surgery ≥ 84 days were independent predictors of stage ypT0–1N0 after nCRT in LARC.

## Data Availability

The data used to support the findings of this study are available from the corresponding author upon request.
